# The Post-COVID-19 Haul on Pulmonary Function: A Prospective Cross-Sectional Study

**DOI:** 10.7759/cureus.61101

**Published:** 2024-05-26

**Authors:** Nitish M S, Revadi G, Ananyan Sampath, Ramesh Gadwala, Charan G V, Abhijit P Pakhare, Rajnish Joshi, Abhishek Singhai, V N Mishra, Sagar Khadanga

**Affiliations:** 1 Internal Medicine, All India Institute of Medical Sciences, Bhopal, Bhopal, IND; 2 Community and Family Medicine, All India Institute of Medical Sciences, Bhopal, Bhopal, IND; 3 Medical School, All India Institute of Medical Sciences, Bhopal, Bhopal, IND; 4 Nephrology, Osmania Medical College, Hyderabad, IND; 5 General Medicine, All India Institute of Medical Sciences, Bhopal, Bhopal, IND

**Keywords:** coronavirus, pulmonary function test, long covid syndrome, respiratory function tests, covid-19, post-acute covid-19 syndrome

## Abstract

Background: Long COVID syndrome, characterized by symptoms like dyspnea, fatigue, and cough, persisting for weeks to months after the initial SARS-CoV-2 infection, poses significant challenges globally. Studies suggest a potential higher risk among females aged 40-50, with symptoms affecting individuals regardless of initial COVID-19 severity, underscoring the need for comprehensive understanding and management.

Methods: A prospective longitudinal study was conducted at a teaching tertiary care institute in Central India, involving COVID-19 patients from May 2020 to September 2021. Participants, aged 18 or older, diagnosed with COVID-19 and surviving until the last follow-up, were monitored telephonically and during outpatient visits for treatment details and outcomes. Data analysis was done using R software 4.2.1.

Results: The baseline characteristics of the study participants showed a majority of moderate COVID-19 severity (47.5%), with a higher proportion of males (64.8%) affected. Common comorbidities included diabetes (27.1%) and hypertension (22.9%). Long COVID-19 symptoms, notably breathlessness, were prevalent, with females exhibiting a significantly higher association. Pulmonary function abnormalities were associated with both long COVID-19 symptoms and higher COVID-19 severity categories, indicating lasting respiratory impact post-infection.

Conclusion: Long after the pandemic, COVID-19 continues to raise concerns due to persistent sequelae, with a majority experiencing long COVID symptoms, particularly those with severe initial illness, including breathlessness and abnormal lung function, highlighting prevalent restrictive lung pattern changes.

## Introduction

The coronavirus disease 2019 (COVID-19), caused by the highly contagious severe acute respiratory syndrome coronavirus 2 (SARS-CoV-2), has had a profound global impact, resembling the scale of the 1918 influenza pandemic. Originating in Wuhan, China, in late 2019, it swiftly spread worldwide, prompting the World Health Organization (WHO) to declare it a pandemic in March 2020. As of September 30, 2022, the WHO reported over 614 million confirmed cases and 6.5 million fatalities globally, with India alone accounting for over 44 million cases and 528,000 deaths. This virus belongs to the family of coronaviruses, initially identified in 1937, known for their distinctive crown-shaped viral particles [1]. While some coronaviruses cause mild upper respiratory illnesses, others like SARS-CoV and MERS-CoV have led to severe outbreaks [1-3].

SARS-CoV-2 has demonstrated its ability to affect multiple organ systems, leading to complications ranging from lung fibrosis to myocardial infarction and neurological issues and from acute to chronic illness states [4,5]. Long after recovering from the initial stages of COVID-19, a considerable number of individuals infected with SARS-CoV-2 continue to experience symptoms. Globally, healthcare practitioners have coined terms like "long-haul COVID-19" or "long-term COVID-19" to refer to these persistent symptoms. Specifically, "long-term COVID-19" denotes individuals who, even after several weeks (typically two to three weeks), have not fully recuperated from SARS-CoV-2 infection [6]. These post-COVID-19 symptoms, often referred to as post-COVID-19 conditions (PCC) or extended COVID-19, manifest in various forms and have led to the usage of multiple terms, such as long COVID-19, long-haul COVID-19, post-acute COVID-19, and post-acute sequelae of SARS-CoV-2 infection (PASC), among others [6,7].

Symptoms may emerge at least four weeks post-infection, with most COVID-19 patients recovering within days to weeks after the initial infection [7]. Studies have highlighted the prevalence of persistent symptoms, such as dyspnea, fatigue, and cough, along with abnormalities in pulmonary function tests (PFTs) even months after the onset of COVID-19 illness [8-10]. These chronic symptoms can affect individuals regardless of the severity of their initial COVID-19 illness [9]. Moreover, studies have indicated a potentially higher risk of long-term COVID-19 among females, particularly between the ages of 40 and 50, with women having double the likelihood of acquiring long-term symptoms compared to males in this age group [11-13]. Although many studies have been published on long-term COVID-19, evidence is scarce in the Indian context. Hence, the present study aims to estimate the prevalence of the long COVID syndrome among participants admitted with acute COVID-19 infection after their discharge through follow-up and identify and line list post-COVID-19 symptoms among prior hospitalized patients six to nine months after discharge from the hospital through tele-follow-up. Second, the study also aims to identify the pulmonary reserves among the patients attending the COVID-19 follow-up clinic.

## Materials and methods

Study setting and design

A prospective longitudinal study design was conducted in a hospital-based setting, where patients were followed up between May 2020 and September 2021 (17 months) by the Department of General Medicine and Pulmonary, Tuberculosis, and Sleep Medicine of All India Institute of Medical Sciences Bhopal, a teaching tertiary care institute in Central India. During the study period, the hospital was designated a COVID-19 treatment center, where in-patients were treated as per (Indian) National Guidelines for COVID-19.

Study Population

This study primarily involved participants over 18 years of age admitted to the COVID ward between May 2020 and October 2020 who had confirmed positive COVID-19 tests as recorded on the admission slip. Exclusion criteria include those who did not survive, whose contact number could not be retrieved, those who were found to have the wrong phone number or whose phone was disconnected, and those who could not be telephonically followed up.

Study Methods

Patient demographic details were recorded at the time of admission. Their treatment information, comorbidities, drugs and dosages administered, and hospital stay outcomes were recorded during their treatment. Patients were followed up telephonically twice a week and were assessed during follow-up visits in outpatient settings.

Ethics statement

This study was designed per the Indian Council of Medical Research (ICMR) ethics guidelines and approved by the Institutional Human Ethics Committee vide IHEC-PGR ref. no. 2020/PG/July/18. Informed consent was obtained from all participants and included in the final analysis.

Sample size calculation

To determine the required sample size for our study, we performed a calculation based on the anticipated prevalence of pulmonary abnormalities in the population with acute COVID-19. We estimated that 15% of this population would exhibit pulmonary abnormalities, with a margin of error of ±5%. Our target population was 1,000,000 individuals, assuming a design effect (DEFF) of 1 for a simple random sample. We calculated the sample size necessary to achieve various confidence levels. For a 95% confidence level, the required sample size was determined to be 196. About 1,010 telephonic consultations were obtained out of the 2663 patients admitted with COVID-19 during the study period. It was expected that 50% of patients might not be contactable for various reasons (insufficient record/non-functional mobile number, etc.). A census-based sampling method was used for the initial line listing of participants. With attrition of 40%, about 612 patients were interviewed telephonically, and about one-third, i.e., 242 cases, were interviewed physically in the COVID-19 Follow-up Clinic. From the same, a consecutive sampling technique was used to randomly select the first 196 patients from those who showed up for physical follow-up. Of the patients who presented to the OPD, 193 patients underwent spirometry, 10 did not give consent, 10 were lost to follow-up, and 30 patients whose test could not be done due to technical difficulties. Data from the modified Medical Research Council (MRC) was available for 192 patients, as one patient’s data was missing. Blood oxygen saturation (SpO_2_) data at rest was available for 178 patients, and COVID-19 severity grading was available for 183 patients.

Statistical analysis

All patient data were abstracted from a standardized data-collection form into a Microsoft Excel Spreadsheet for cleaning before analysis. All data analysis was performed on R software version 4.2.1 (R Core Team, R: A language and environment for statistical computing, R Foundation for Statistical Computing, Vienna, Austria). The data were summarized as frequencies or percentages for categorical variables and as means and standard deviations or median and interquartile ranges for continuous variables, depending on the distribution. Independent variables were assessed for association with mMRC and WHO COVID-19 severity grading using the Kruskal-Wallis test. P value <0.05 was considered significant.

Data visualization was done using a box violin jitter plot, a hybrid plot consisting of a violin plot, jitter plot, and box whisker plot. With the X-axis showing the Modified Medical Research Council (mMRC) grades of breathlessness in Figure 2, COVID-19 severity categories in Figure 3 and values of forced expiratory volume in the first second (FEV1), forced vital capacity (FVC), FEV1 per cent predicted, and FVC per cent predicted along the Y axis. The mean values of FEV1, FVC, FEV1 per cent predicted, and FVC per cent predicted in each mMRC grade of breathlessness categories were plotted as dots in the median of the box whisker plot. Welch ANOVA F test was used to compare the mean between the various MMRC grades of breathlessness and WHO COVID-19 categories. The Games-Howell test was used to compare the difference between pairwise group means and show whether differences were significant or not.

Operational Definitions

COVID-19 confirmed case: A person with a positive nucleic acid amplification test (NAAT), regardless of clinical criteria or epidemiological criteria or a person meeting the clinical criteria and /or epidemiological criteria with a positive professional-use or self-test SARS-CoV-2 antigen-RDT [14].

Long COVID symptoms: COVID-19-related new, recurrent, or persistent symptoms (Nalbandian, March 2021; Hope, June 2022) and health issues that develop four weeks or more after an acute COVID-19 infection are referred to as "post-COVID conditions"/“long COVID symptoms” by the US Centers for Disease Control and Prevention [15,16].

Obstructive pattern: Spirometry values with FEV1/FVC <0.7 [17].

Restrictive pattern: FVC < lower limit of normal or <80% expected FVC [18].

The Medical Research Council Dyspnea scale was used to assess self-reported dyspnea by patients, as shown in Table 1 [19].

**Table 1:** Self-described dyspnea score as per the Modified Medical Research Council (mMRC)

mMRC reported grade	Subjective description equivalent to the reported grade
0	Breathless with strenuous exercise.
1	Shortness of breath when hurrying on level ground or walking up a slight hill.
2	On level ground, I walk slower than people of the same age because of breathlessness, or have to stop for breath when walking at my own pace.
3	I stop for breath after walking about 100 yards or after a few minutes on level ground.
4	I am too breathless to leave the house or I am breathless when dressing.

The WHO COVID-19 severity score was used to clinically categorize patients based on signs and symptoms, as shown in Table 2 [20].

**Table 2:** WHO COVID-19 clinical categorization

WHO COVID-19 severity	Clinical implication of grade
0	Mild: patients with symptoms who meet the COVID-19 case definition but do not have any signs of viral pneumonia or hypoxia
1	Moderate: no signs of severe pneumonia, including SpO2 90% on room air, but does show clinical signs of pneumonia (fever, cough, dyspnoea, fast breathing)
2	Severe: with one of the following along with the clinical symptoms of pneumonia (fever, cough, and dyspnoea) respiratory rate > 30 breaths/min; severe respiratory distress; or SpO2 < 90% on room air
3	Critical: sepsis, septic shock, and acute respiratory distress syndrome (ARDS)

## Results

**Figure 1:**
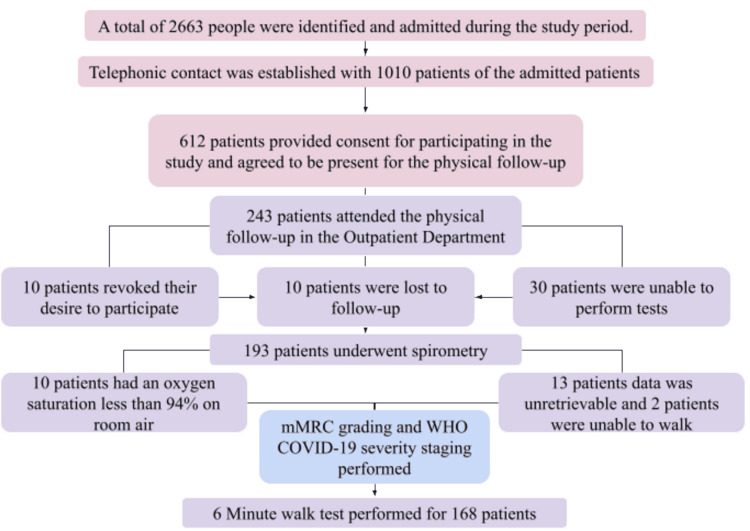
Flow diagram showing study population recruitment and assessment for various parameters.

Among the 193 total participants recruited in the study, 125 participants (64.7%) reported some form of long-term COVID-19 syndrome. As COVID-19 posed a significant burden on the healthcare personnel, patient follow-up and data management were performed in less-than-ideal settings. The patient recruitment algorithm is shown in Figure 1.

Table 3 depicts the baseline characteristics of all participants included in the study along with the number of participants for whom each data variable was available. Most of the patients were of moderate severity (87, 47.5%), followed by mild (65, 35.5%), severe (19, 10.4%), and critical (12, 6.6%) severity. Out of 193 participants, 125 (64.8%) were males with a median (IQR) age group of 46.0 (35.0, 56.0). The most common comorbidity among the 28 participants who reported comorbidity was diabetes 13 (27.1%), followed by hypertension (11, 22.9 %). 

**Table 3:** Participant distribution of the baseline demographics and clinical characteristics. 1: median (IQR), n (%)

Characteristic	Overall, N	n
Demographic profile^1^		
Age (years)	192	46.0 (35.0, 56.0)
Sex	193	
Female		68 (35.2%)
Male		125 (64.8%)
Weight (kg)	184	70.0 (62.0, 80.0)
Height (cms)	180	165 (157.0, 170.2)
Duration of hospital stay (days)	152	10.0 (6.0, 15.0)
Participants having comorbidities at the time of hospitalization		
No known comorbidities		165 (85.5%)
Presence of comorbidities	28	28 (14.5%)
Type 2 diabetes mellitus		13 (27.1%)
Hypertension		11 (25.0%)
Stroke		2 (4.0%)
Chronic kidney disease		1 (2.1%)
Chronic obstructive pulmonary disease		1 (2.3%)
mMRC grading of dyspnea		
0	192	123 (64.1%)
1		35 (18.2%)
2		15 (7.8%)
3		18 (9.4%)
4		1 (0.5%)
WHO COVID-19 severity		
0- mild	183	65 (35.5%)
1- moderate		87 (47.5%)
2- severe		19 (10.4%)
3- critical		12 (6.6%)

Among the various types of long COVID-19 symptoms documented among the study participants at the follow-up, breathlessness was the most common with 64 (33.2%), followed by fatigue (45, 16.4%), cough (12, 4.8%), arthralgia (11, 4.8%), and myalgia (9, 4.7%). While comparing the various long COVID-19 symptoms by sex, it was found that being a female (53, 77.9%) was associated with a higher chance of having long-COVID syndromes as compared to being a male participant, which was significantly associated with long COVID-19 symptoms compared to males (72, 57.6%) (p = 0.002). Among the various symptoms, fatigability (p = 0.004) and throat irritation (p = 0.042) were significantly associated with the female sex. Among 125 patients who had long COVID-19 symptoms, 34 (7.2% ) had more than one long COVID-19 symptom at the time of follow-up. All patients complaining of breathlessness were assessed for severity by mMRC grading of breathlessness. There were 64 (32.2%) patients who complained of having breathlessness, and the majority of 35 (18.2%) had grade 1 breathlessness in mMRC. Table 4 shows the distribution of pulmonary function testing and six-minute walk testing on the participants stratified by their mMRC grades.

**Table 4:** Distribution of the pulmonary function testing parameters, six-minute walk test, and oxygen saturation versus mMRC grading and WHO COVID-19 category p < 0.05 was considered significant.

Characteristic	mMRC grade Mean (SD)						WHO COVID-19 grade Mean (SD)						
	Number	Overall, N = 192	0, N = 123	1, N = 35	2 to 4, N = 34	p- value	Number	Overall, N = 183	0, N = 65	1, N = 87	2 to 3, N = 31	p- value	
													PFT													
FEV1 (liters)	192	2.1 (1.6, 2.7)	2.5(1.8,2.9)	2.0 (1.6,2.3)	1.6 (1.2,1.8)	<0.001	183	2.1 (1.6, 2.7)	2.6 (2.1, 2.9)	1.8 (1.5, 2.7)	1.7 (1.5,2.1)	<0.001	
													FEV1% predicted	72.0 (57.5,84.5)	77.0 (67.0,87.0)	66(54.0,73.5)	61(44.0,76.5)	<0.001	71.0 (57.0, 84.0)	80.5 (69.0,87.0)	70.0 (54.5,80.5)	61.5 (46.0,71.0)	<0.001	
FVC (liters)		2.6 (1.9, 3.3)	2.8 (2.2,3.5)	2.3 (1.9,2.7)	1.8 (1.5,2.2)	<0.001		2.6 (1.9, 3.3)	3.0 (2.6, 3.6)	2.4 (1.7, 3.3)	2.0 (1.6,2.5)	<0.001												
																								FVC% predicted	73.0 (58.5,83.5)	76.0 (66.0,87.0)	66(51.0,77.0)	56(37.0,77.0)	<0.001	72.0 (58.0, 84.0)	83.0 (71.0,88.0)	71.0 (54.0,80.0)	58.5 (46.2,65.8)	<0.001	
FEV1/FVC		85.2 (79.9,89.2)	85.2 (81.0,88.8)	85.6 (79.7,88.8)	83.7 (78.2,93.3)	0.991		85.2 (80.0, 89.2)	83.7 (78.0,87.9)	85.0 (79.9,89.2)	88.6 (85.9,95.3)	<0.001																						
													PFT Interpretation													
													Normal n(n%)	192	131 (68.2%)	96 (78%)	20 (57.1%)	15 (44.0%)	<0.001	183	124 (67.8%)	53(81.5%)	54 (62.1%)	17 (54.8%)	0.002	
Obstructive n(n%)		10 (5.2%)	6 (4.9%)	2 (5.7%)	2 (5.9%)			10(5.5%)	4 (6.2%)	6(6.9%)	0 (0.0%)														
Restrictive n(n%)		49 (27.1%)	21 (17.1%)	13 (37.1%)	16 (47.0%)			48 (26.8%)	7 (10.8%)	27(31.0%)	14(45.2%)														
Mixed n(n%)		1 (0.6%)	0 (0.0%)	0 (0.0%)	1 (3.1%)			1 (0.5%)	1 (1.5%)	0 (0.0%)	0 (0.0%)														
spO2% at rest		178	97 (96.0,98.0)	98 (97.0,98.0)	97 (96.0,98.0)	96 (95.0,97.0)		<0.001	170	97.0 (96.0,98.0)	98.0 (97.0,99.0)	97.0 (96.0,98.0)	97.0 (96.0,98.0)		0.002										
														Six-minute walking test (6MWT)													
6MWT distance (meters)	168	360 (271.2,412.5)	395 (330.0,430.0)	350 (175.0,400)	150 (52.2,327.5)	<0.001	159	360.0 (255.0, 410.0)	400.0 (350.0, 431.0)	350.0 (162.5, 400.0)	320 (150.0,397.5)	<0.001	
													6MWT status N = 168													
Completed n(n%)		125(74.4%)	96 (85.0%)	20 (64.5%)	9 (37.5%)	<0.001	159	117 (73.6%)	51 (83.6%)	54 (72.0%)	12 (52.2%)	0.013	
													Terminated n(n%)		43 (25.6%)	17 (15.0%)	11 (35.5%)	15 (62.5%)	42 (26.4%)	10 (16.4%)	21 (28.0%)	11 (47.8%)	
Abnormal 6MWT n(n%)	164	137 (71.3%)	94 (76.4%)	23 (65.71%)	20 (58.8%)			0.332	156	133(72.67%)	49(75.38%)												65(75.38)	19(61.29%)	0.602	

**Figure 2:**
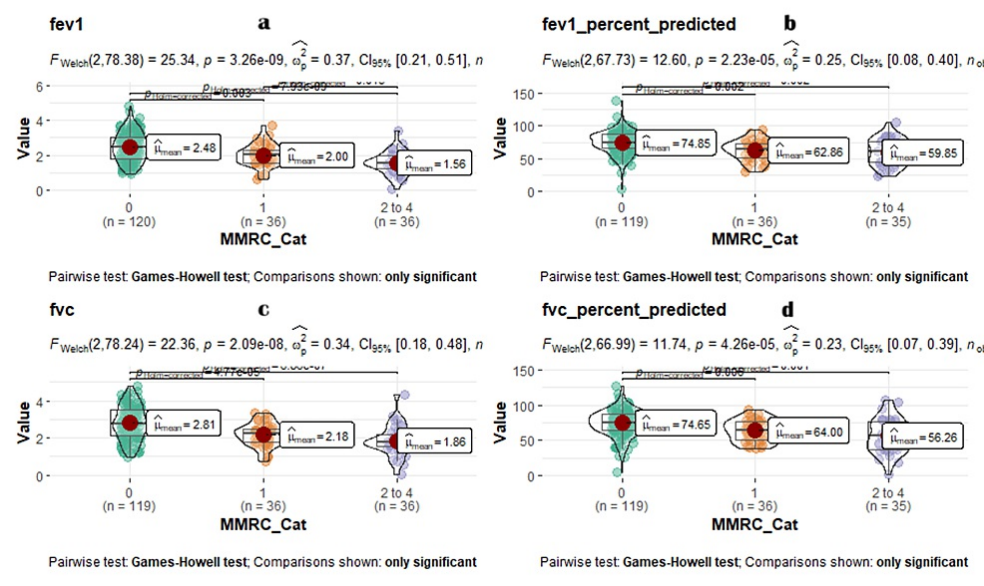
Comparison violin plot comparing PFT parameters with mMRC grades. X-axis: mMRC grades, Y-axis: a) FEV1, b) FEV1 percent predicted, c) FVC, and d) FVC percent. MMRC grade 0 (n =120, coded in green round dots), MMRC grade 1 (n =36, coded in orange round dots), MMRC grade 2 to 4 (n = 36, coded in purple round dots). a) Left-top image shows the FEV1 values. b) Right-top image shows the FEV1 percent predicted values. c) Left bottom shows the FVC values. d) Right bottom shows the FVC percent predicted values along the y-axis with all their mean values as red dots.

The FEV1, FEV1% predicted, FVC, and FVC% predicted had a significant association (p < 0.001) with higher grades of MMRC. Among the 49 (27.1%) participants who presented with a restrictive pattern of the PFT, 21 (17.1%) of the population had mMRC grade 0, 13 (37.1%) had grade 1, and 16 (47.0%) had grades 2-4 of breathlessness. Almost half (44.1%) of the participants presented with MMRC grade 2-4 versus 37.1% with MMRC grade 1 versus 17.1% with MMRC grade 0. Among 5.2% of the participants who had a restrictive pattern of the PFT, 18.2% presented with MMRC grades 2-4 versus 18.2% with MMRC grade 1 versus 54.5% with MMRC grade 0 of breathlessness. A graphical representation of all parameters with mMRC grade is shown in Figure 2.

The median distance traveled by each grade of breathlessness progressively decreased as the grades of breathlessness increased: 395 meters in grade 0, 359 meters in grade 1, and 150 meters in grades 2-4, indicating a significant association (p < 0.001). The decreasing trend of the median baseline SpO_2_ % was 98%, 97%, and 96%, respectively, for worsening mMRC grades 0, 1, and 2-4 of breathlessness, which was statistically significant (p < 0.001). The percentage of participants who had to terminate 6MWT because of a fall in SpO_2_ was 17 (39.5.15%), 11 (35.525.58%), and 15 (34.8862.5%), respectively, for grades 0, 1, and 2-4 mMRC, for worsening breathlessness, which was statistically significant (p < 0.001). The restrictive and obstructive patterns of the PFT showed an association with increasing grades of mMRC severity, which was statistically significant (p < 0.001). 

**Figure 3:**
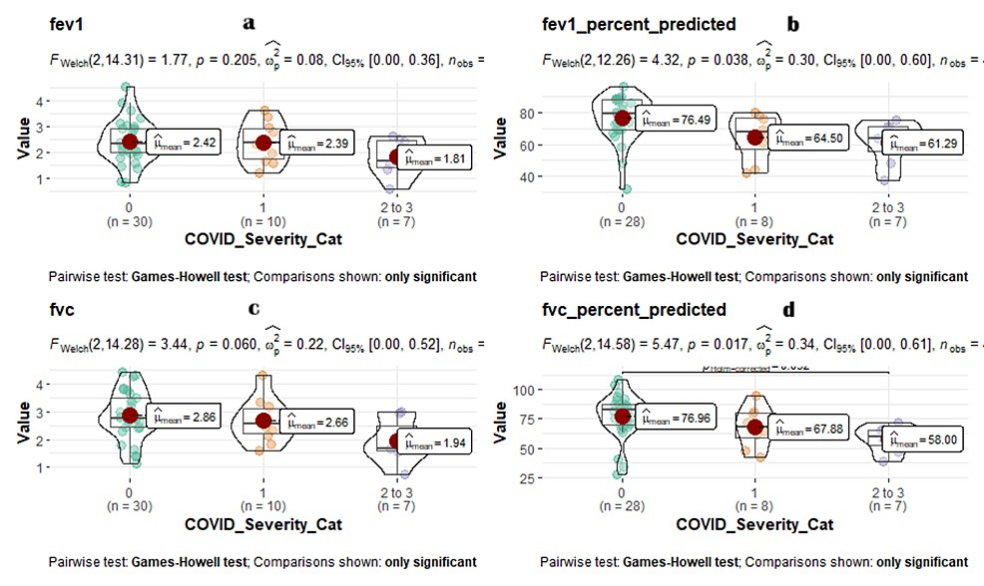
Comparison violin plot comparing PFT parameters with COVID-19 severity grades. X-axis: WHO COVID-19 severity grades, Y-axis: a) FEV1, b) FEV1 percent predicted, c) FVC, and d) FVC percent. COVID-19 severity category 0 (n = 30, coded in green round dots), COVID-19 severity category 2 (n = 10, coded in green round dots), and COVID-19 severity categories 3-4 (n = 7, coded in green round dots). a) Left-top image shows the FEV1 values. b) Right-top image shows the FEV1 percent predicted values. c) Left bottom shows the FVC values. d) Right bottom shows the FVC percent predicted values along the y-axis with all their mean values as red dots.

The FEV1, FEV1% predicted, FVC, and FVC% predicted had a significant association (p < 0.001) with higher grades of COVID-19 severity. Among the 48 (26.87%) participants who presented with a restrictive pattern of the PFT, seven (14.58%) presented with COVID category 0, 27 (56.25%) presented with category 1, and 14 (29.1%) with category 2-3 (p 0.002). Almost half (44.1%) of the participants presented with COVID-19 severity in categories 2 and 3 versus 29.1% in category 1 versus 14.6% in category 0. Among 5.2% of participants who had a restrictive pattern of PFT, 60% presented with COVID-19 severity category 1 versus 40% with category 0. The restrictive and obstructive patterns of PFT showed an association with increasing grades of COVID-19 severity, which was statistically significant (p = 0.002). Figure 3 shows the distribution of PFT parameters against various WHO COVID-19 grades as a box violin plot, with significant pairwise comparisons.

## Discussion

Post-COVID sequelae continue to cause significant distress to individuals beyond their acute COVID-19 syndrome. A majority of these symptoms are often overlooked or attributed to other minor illnesses, not associated to the acute COVID-19 stage. This study was designed to estimate the prevalence of this phenomenon among individuals in central India and hence also evaluate their pulmonary function as a proxy for the residual effects of COVID-19.

Our study involved a population majorly suffering from no comorbidities at a median age of 46 years with two-thirds of the participants being males. This gender distribution was consistent with findings from Patel et al. (2022) in India, Polese et al. (2021) in Brazil, Carfi et al. (2020) in Italy, and Mandal et al. (2023) with a higher proportion of male participation (61%, 73%, 62.9%, and 62%) at a range of 51-65 years, respectively [21-24]. Meanwhile, a more balanced proportion was observed in Dryden et al (2022) in South Africa of nearly 49% males with a median age of 52 years [25]. The median days of hospitalization were 10 days, similar to the findings of Patel et al. (2022), who reported a median duration of 11 days for hospitalization in their study [21]. Among our study, participants who had comorbidities, diabetes, and hypertension were the most common. This pattern aligns with observations by Patel et al. (2022), Polese et al. (2021) in Brazil, Carfi et al. (2020) in Italy, Mandal et al. (2023) in India, and Dryden et al. (2022) and Kim et al. (2022) in South Korea as prevalent comorbidities in their cohort [21-26].

A majority of the participants experienced persistent symptoms, with breathlessness being the most common, followed by fatigue, cough, arthralgia, and myalgia. These findings are concordant with prevailing literature as reported by Mandal et al. (2021) from India and Polese et al. (2021) from Brazil, emphasizing the prevalence of breathlessness and fatigue as prominent long COVID symptoms [22,24]. In our study, nearly a third of patients had abnormal PFTs, predominantly showing a restrictive pattern. This aligns with previous research showing varying degrees of pulmonary function abnormalities in post-COVID patients [9,26]. Reports suggest that a significant proportion of COVID-19 infections, ranging from 14% to 19%, result in cardiac damage, with proposed causes including direct cardiac injury, downregulation of ACE2 receptors, severe inflammation, and hypoxia damage as other aspects of long COVID syndrome not discussed or observed in our study [6-8,16,23,27]. 

The 6MWT revealed abnormalities in patients, with no sex-based differences observed. In addition, there was a significant association between PFT parameters, 6MWT results, and mMRC grades. This indicates the impact of COVID severity on long-term pulmonary function and exercise capacity in individuals with the long COVID syndrome. Comparing PFTs with COVID severity categories at prior hospitalization, we found a significant association between abnormal spirometry tests and increasing COVID severity categories. Furthermore, decreased 6MWT distance was associated with higher COVID severity categories, highlighting the lasting effects of severe COVID-19 illness on physical function. Furthermore, investigations into post-COVID-19 pulmonary function have revealed a high prevalence of abnormalities such as restrictive patterns and decreased lung capacity among survivors. The impact of COVID-19 on respiratory function persists for extended periods, with a significant number of patients experiencing symptoms and impaired lung function even several months after recovery.

Overall, our study adds to the growing body of evidence on the prevalence and impact of long-term COVID-19, emphasizing the need for continued monitoring and comprehensive care for post-COVID patients, especially those with severe illness and persistent symptoms. This study analyzes the impact of COVID-19 beyond its acute presentation in terms of objective and subjective self-reported parameters, hence analyzing the determinants of the sequelae. This study analyzes the impact of COVID-19 both as objective and subjective parameters, thereby adding to the strength of its results. Our study has several limitations. Only about 7% (193 of 2663) of patients who were admitted for COVID-19 were followed up physically in the study. The baseline PFT was unavailable and could not be compared. There is missing data even among the various variables due to the restricted data recording abilities during the COVID-19 pandemic. However, our study is a real-life scenario during the unprecedented COVID-19 pandemic. The study also observed a large attrition rate of 40% of individuals from the original sample who could not be contacted telephonically.

## Conclusions

COVID-19 has remained a disease of concern beyond the pandemic for its persistent sequelae, as evidenced by a six-month follow-up in our study. A majority of the population suffered from long COVID syndromes, both in terms of subjective breathlessness and abnormal pulmonary function tests, with it being more consistently found among participants with higher grades of COVID-19 severity at presentation. This study also emphasizes the associated prevalence of restrictive pattern lung changes in patients with long COVID-19 syndrome among the study population.
